# Arytenoideraphy

**Published:** 1898-08

**Authors:** 


Arytenoideraphy. Arytenoideraphy is a new surgical treatment for roaring due to laryngeal hemiplegia. The operation, *born* and *christened* during the clinics of 1897–1898, consists of excision of the vocal cord and suturing the arytenoid cartilage to the crico-thyroidean ligament. Six patients have received the treatment to date with results varying from marked improvement to complete recovery.

From a physiological and surgical aspect it is more rational than arytenectomy, and being much more simple is not followed with the same serious sequelæ.

A series of experiments is now under way in the department of surgery, and a detailed description will be submitted to the profession as soon as sufficient data is at hand.

